# Supplementary material for: Erwinia phage Asesino is a nucleus-forming phage that lacks PhuZ
Source: Sci Rep. 2025 Jan 11;15:1692. doi: 10.1038/s41598-024-64095-4 (PMC11724907; doi:10.1038/s41598-024-64095-4)
Supplement: Supplementary file 1 — Supplementary Information. [file 41598_2024_64095_MOESM1_ESM.pdf]

Supplementary Information

Table S1. All phages used in phylogenetic analysis figures. The common name, binomal name, NCBI accession number, and whether the available genome sequence is published as a complete or partial genome are listed. For the officially recognized phages, their binomial names are listed as they are in the ICTV taxonomy browser. For unclassified phages, their unofficial classification as listed in the NCBI taxonomy browser was used to fill in this information, if available, and marked with an asterisk in this table.

Phage Name	Binomial Classification (if available)	Genome Accession	Sequence Completeness
Aeromonas phage AerS_266	unclassified *Chaoshanvirus**	OR496884	Complete Genome
Aeromonas phage ZPAH34	*Chaoshanvirus ZPAH34*	NC_071007	Complete Genome
Aeromonas phage CF8	unclassified *Chaoshanvirus**	MK774614	Complete Genome
Aeromonas phage pAEv1810	*Ferozepurvirus pAEv1810*	OL964756	Complete Genome
Aeromonas phage PS1	*Ferozepurvirus PS1*	MN032614	Complete Genome
Aeromonas phage D6	*Ludhianavirus D6*	MN131137	Complete Genome
Aeromonas phage D3	*Ludhianavirus D3*	MN102098	Complete Genome
Aeromonas phage LAh10	*Ludhianavirus LAh10*	MK838116	Complete Genome
Serraria phage vB_SmaM-Yubaba	unclassified *Petsuvirus**	ON287375	Complete Genome
Klebsiella phage N1M2	*Nimduovirus N1M2*	MN642089	Complete Genome
Pseudomonas phage OBP	unclassified *Petsuvirus**	JN627160	Complete Genome
Edwardsiella pEt-SU	*Petsuvirus pEtSU*	NC_048182	Complete Genome
Erwinia phage AH04	*Meadowvirus AH04*	MZ501267	Complete Genome
Kosakonia phage Kc263	*Branisovskavirus Kc263*	MZ348422	Complete Genome
Photobacterium phage PDCC-1	*Aphroditevirus PDCC1*	NC_048821	Complete Genome
Vibrio phage 2 TSL-2019	*Aphroditevirus av2TSL2019*	NC_048747	Complete Genome
Vibrio phage USC-1	*Aphroditevirus USC1*	NC_048779	Complete Genome

Vibrio phage Aphrodite1	*Aphroditevirus aphrodite1*	NC_042100	Complete Genome
Vibrio phage VP4B	*Tidunavirus VP4B*	NC_042136	*Partial Genome*
Vibrio phage pTD1	*Tidunavirus pTD1*	NC_041916	Complete Genome
Vibrio phage phiKT1028	unclassified *Tidunavirus**	OP123707	Complete Genome
Klebsiella phage Miami	*Miamivirus miami*	MT701590	Complete Genome
Klebsiella phage phiKp_32	N/A	LC768500	Complete Genome
Erwinia phage vB_EamM_RisingSun	*Risingsunvirus risingsun*	NC_042018	Complete Genome
Pseudomonas phage EL	*Elvirus EL*	NC_007623	Complete Genome
Shewanella phage FishSpeaker	N/A	OP589283	Complete Genome
Erwinia phage vB_EamM_Simmy50	*Agricanvirus simmy50*	NC_041974	Complete Genome
Erwinia phage vB_EamM_RAY	*Agricanvirus ray*	NC_041973	Complete Genome
Erwinia phage vB_EamM_Special G	*Agricanvirus specialG*	NC_041975	Complete Genome
Erwinia phage vB_EamM_Desertfox	*Agricanvirus desertfox*	NC_042098	Complete Genome
Erwinia phage vB_EamM_Deimos-Minion	*Agricanvirus deimos*	NC_041972	Complete Genome
Erwinia phage Ea35-70	*Agricanvirus Ea3570*	NC_023557	Complete Genome
Erwinia phage AH06	unclassified *Agricanvirus**	MZ501268	Complete Genome
Erwinia phage pEa_SNUABM_37	N/A	MW845760	Complete Genome
Salmonella phage vB_SalM_SA002	N/A	MN445183	Complete Genome
Proteus phage 10	N/A	MT661596	Complete Genome
Escherichia phage vB_EcoM_Goslar	*Goslarvirus goslar*	NC_048170	Complete Genome
Serraria phage Moabite	*Moabitevirus moabite*	NC_048792	Complete Genome

Serraria phage vB_SmaM_ 2050HW	*Moabitevirus mv2050HW*	NC_048668	Complete Genome
Serraria phage vB_SmaS-Totoro	unclassified *Moabitevirus**	ON287372	Complete Genome
Klebsiella phage KpLz-2_45	*Siatvirus Lz245*	NC_061418	Complete Genome
Klebsiella phage vB_KpM_FBKp24	*Maaswegvirus Kp24*	MW394391	Complete Genome
Erwinia phage vB_Ea277G	unclassified *Iapetusvirus**	OP991903	Complete Genome
Erwinia phage PhiEaH1	*Iatpetusvirus EaH1*	NC_023610	Complete Genome
Serraria phage PCH45	N/A	MN334766	Complete Genome
Erwinia phage pEa_SNUABM_54	N/A	MW879341	Complete Genome
Klebsiella phage vB_KvM-Eowyn	*Eowynvirus eowyn*	LR881104	Complete Genome
Pseudomonas phage SL2	*Phikzvirus SL2*	NC_042081	Complete Genome
Pseudomonas phage PA7	*Phikzvirus PA7*	MZ444140	Complete Genome
Pseudomonas phage phiKZ	*Phikzvirus phiKZ*	NC_004629	Complete Genome
Pseudomonas phage PS119XW	*Pawinskivirus PS119XW*	MK599315	Complete Genome
Pseudomonas phage PhiPA3	*Miltoncavirus PhiPA3*	HQ630627	Complete Genome
Pseudomonas phage Phabio	*Phabiovirus phabio*	MF042360	Complete Genome
Pseudomonas phage 201phi2-1	*Serwervirus 201phi21*	EU197055	Complete Genome
Pseudomonas phage D6	N/A	OQ363659	Complete Genome
Pseudomonas phage Psa21	*Tepukevirus Psa21*	MK552327	Complete Genome
Pseudomonas phage Noxifer	*Noxifervirus noxifer*	NC_041994	Complete Genome
Pseudomonas phage pPa_SNUABM_DT01	N/A	MW735835	Complete Genome
Ralstonia phage RSL2	*Chiangmaivirus RSL2*	NC_028950	Complete Genome

Burkholderia phage FLC6	unclassified *Chiangmaivirus**	LC592711	Complete Genome
Ralstonia phage RSF1	*Chiangmaivirus RSF1*	NC_028899	Complete Genome
Ralstonia phage RP12	*Ripduovirus RP12*	NC_041911	Complete Genome
Burkholderia phage FLC9	N/A	LC667451	Complete Genome
Xanthomonas phage RTH11	N/A	OQ790161	Complete Genome
Xanthomonas phage vB_XciM_LucasX	N/A	MW825358	Complete Genome
Xanthomonas phage Xoo-sp14	N/A	MT939492	Complete Genome
Salmonella phage MET_P1_082_240	unclassified *Seoulvirus**	OQ383623	Complete Genome
Salmonella phage SPN3US	*Seoulvirus SPN3US*	NC_027402	Complete Genome
Proteus phage 7	*Seoulvirus SPN3US**	MT679221	Complete Genome
Enterobacteria phage SEGD1	*Seoulvirus SPN3US**	KU726251	Complete Genome
Salmonella phage SaP7	*Seoulvirus SPN3US**	OM456492	Complete Genome
Escherichia phage vB_EcoM_Lh1B	unclassified *Seoulvirus**	OK428535	Complete Genome
Escherichia phage vB_EcoM_EC001	*Seoulvirus SPN3US**	MN445185	Complete Genome
Salmonella phage JN03	unclassified *Seoulvirus**	MT799840	Complete Genome
Salmonella phage SPAsTU	unclassified *Seoulvirus**	MH221129	Complete Genome
Salmonella phage STsAS	unclassified *Seoulvirus**	MH221128	Complete Genome
Erwinia phage pEa_SNUABM_29	unclassified *Seoulvirus**	MW812339	Complete Genome
Erwinia phage phiEaH2	*Erskinevirus EaH2*	NC_019929	Complete Genome
Erwinia phage vB_EamM_Stratton	*Erskinevirus EaH2**	KX397373	Complete Genome
Erwinia phage vB_EamM_Asesino	*Erskinevirus asesino*	NC_031107	Complete Genome

Erwinia phage vB_EamM_Huxley	*Machinavirus machina**	NC_031127	Complete Genome
Erwinia phage vB_EamM_Parshik	unclassified *Machinavirus**	KX397371	Complete Genome
Erwinia phage Machina	*Machinavirus machina*	NC_042056	Complete Genome
Salmonella phage pSal-SNUABM-04	unclassified *Machinavirus**	MT710307	Complete Genome
Erwinia phage pEa_SNUABM_42	unclassified *Machinavirus**	MW812340	Complete Genome
Erwinia phage pEa_SNUABM_43	unclassified *Machinavirus**	MW384823	Complete Genome
Erwinia phage pEa_SNUABM_10	unclassified *Machinavirus**	MW812342	Complete Genome
Erwinia phage pEa_SNUABM_6	unclassified *Machinavirus**	MW812343	Complete Genome
Erwinia phage vB_EamM_ChrisDB	unclassified *Machinavirus**	NC_031126	Complete Genome
Erwinia phage pEa_SNUABM_9	unclassified *Machinavirus**	MW812341	Complete Genome
Erwinia phage pEa_SNUABM_4	unclassified *Machinavirus**	MW812338	Complete Genome
Erwinia phage pEa_SNUABM_8	unclassified *Machinavirus**	MW760841	Complete Genome
Erwinia phage vB_EamM_Caitlin	unclassified *Machinavirus**	NC_031120	Complete Genome
Erwinia phage Wellington	*Wellingtonvirus wellington*	NC_048016	Complete Genome
Erwinia phage vB_EamM_Kwan	*Wellingtonvirus wellington**	NC_031010	Complete Genome
Erwinia phage pEa_SNUABM_11	unclassified *Wellingtonvirus**	MW845758	Complete Genome
Erwinia phage Derbicus	*Derbicusvirus derbicus*	NC_048173	Complete Genome
Erwinia phage vB_EamM_EarlPhillipIV	*Derbicusvirus derbicus**	NC_031007	Complete Genome
Erwinia phage pEa_SNUABM_38	unclassified *Derbicusvirus**	MW845759	Complete Genome
Cronobacter phage CR5	unclassified *Derbicusvirus**	NC_021531	Complete Genome
Erwinia phage vB_EamM_Phobos	unclassified *Derbicusvirus**	NC_031043	Complete Genome

Vibrio phage pVa-21	N/A	KY499642	Complete Genome
Bacillus phage vB_BspM_AgentSmith	N/A	MW749006	Complete Genome
Vibrio phage JM-2012	N/A	NC_017975	Complete Genome
Vibrio phage vB_VpaM_sm033	N/A	OV032902	Complete Genome
Vibrio phage vB_pir03	N/A	MT811961	Complete Genome
Vibrio phage BONAISHI	N/A	MH595538	Complete Genome
Vibrio phage vB_VmeM-Yong XC31	N/A	MK308674	Complete Genome
Staphylococcus phage SPbeta-like	N/A	NC_029119	Complete Genome
Bacillus phage SPBc2	*Spbetavirus SPbeta*	NC_001884	Complete Genome
Brevibacillus phage Sundance	N/A	NC_028749	Complete Genome
Geobacillus phage E3	N/A	NC_029073	Complete Genome
Clostridium phage c-st	N/A	NC_007581	Complete Genome
Bacillus phage G	*Donellivirus gee*	NC_023719	Complete Genome
Bacillus phage PBS1	*Takahashivirus PBS1*	NC_043027	Complete Genome
Bacillus phage AR9	*Takahashivirus PBS1**	NC_031039	Complete Genome
Staphylococcus phage PALS_2	N/A	MN091626	Complete Genome
Yersinia phage phiR1-37	N/A	NC_016163	Complete Genome

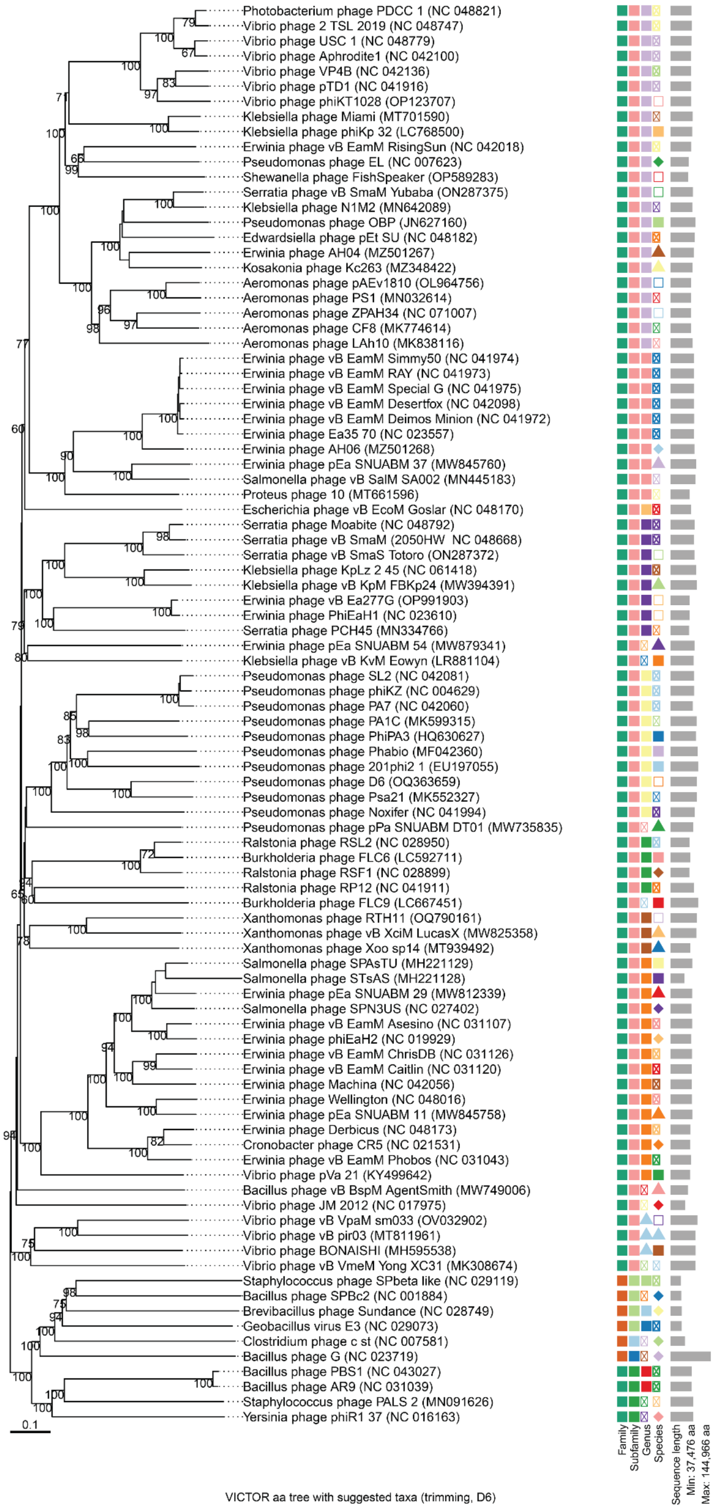

Figure S1. VICTOR Genome Phylogeny Results. Original data from VICTOR[45] showing predicted family, subfamily, genus, and species assignments. As many of these phages are unclassified, we use the tentative genus-level taxonomy provided here to annotate Figure 1. Note: VICTOR-predicted genus assignments do not always agree with accepted taxonomy and are simply used here for color-coding divergent clades of chimalliviruses to improve readability, not for proposing real taxonomic groups.

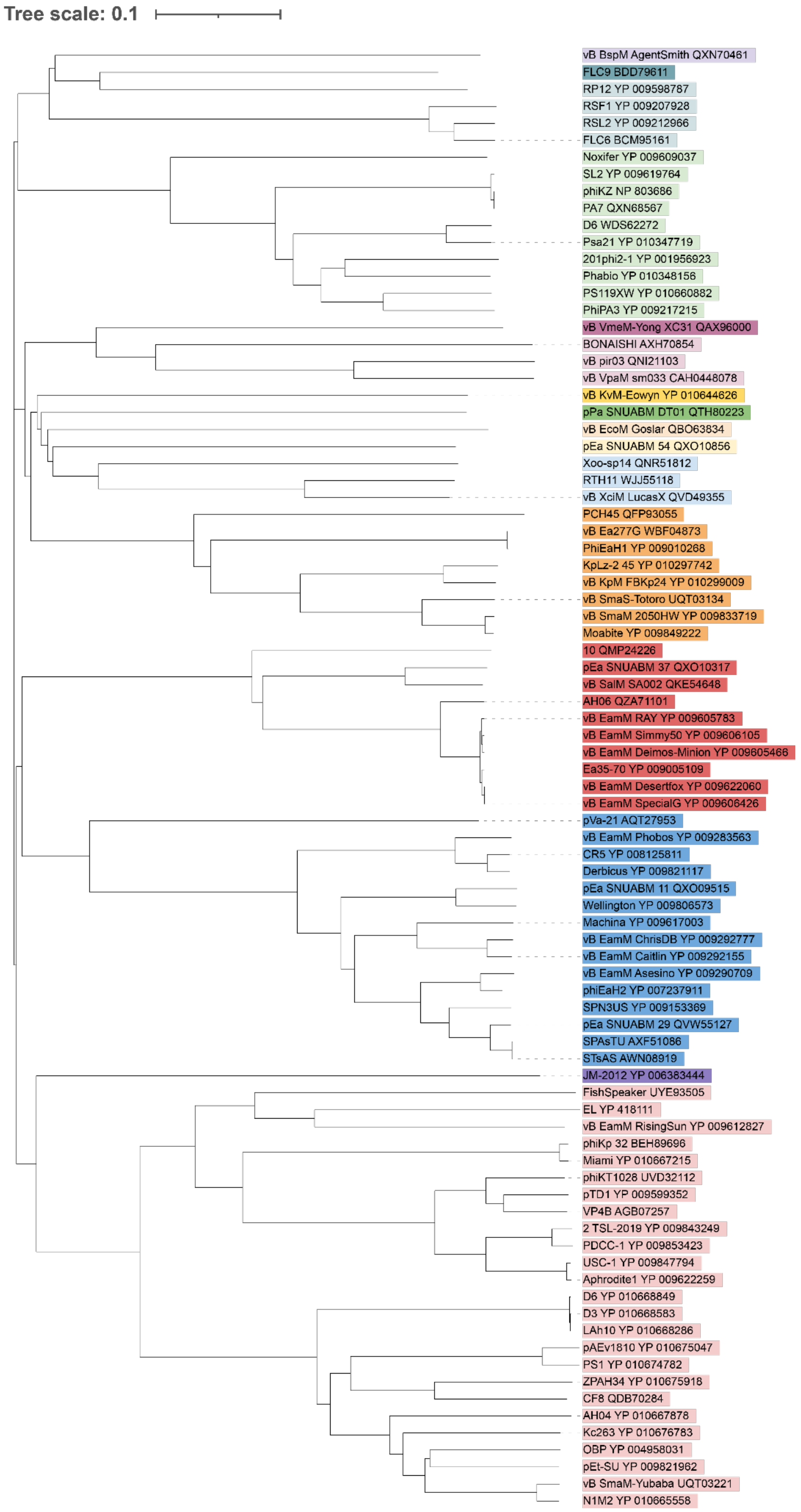

Figure S2. Major Capsid Protein Tree. The same clades of chimalliviruses are seen in a phylogenetic tree made using the major capsid protein multiple sequence alignment as in the whole-genome phylogenetic analysis (Figure 1A). The clades are color-coded to match Figure 1A.
